# T4 rather than TSH correlates with BMD among euthyroid adults

**DOI:** 10.3389/fendo.2022.1039079

**Published:** 2023-01-09

**Authors:** Ning Sheng, Fei Xing, Jie Wang, Xin Duan, Zhou Xiang

**Affiliations:** Orthopedic Research Institute, Department of Orthopedics, West China Hospital, Sichuan University, Chengdu, Sichuan, China

**Keywords:** thyroid hormone, T4, bone mineral density, NHANES, euthyroid

## Abstract

**Purpose:**

The objective of this study was to evaluate the association between thyroid hormone and bone mineral density (BMD) among euthyroid adults.

**Methods:**

This cross-sectional study researched the information from the National Health and Nutrition Examination Survey 2007–2010. We included 3,759 euthyroid participants finally. We used multivariate linear regression models to evaluate the linear relationship between the thyroid hormone profile and BMD. Subgroup analyses stratified by gender and age were further performed. Moreover, the nonlinear relationship was characterized by fitted smoothing curves and generalized additive models, and logistic regression models were used to determine the association of thyroid-stimulating hormone (TSH) and thyroxine (T4) with previous fractures.

**Results:**

The weighted multivariable linear regression models showed no association between TSH and BMD. Free thyroxine (FT4), T4, free triiodothyronine (FT3), and total triiodothyronine (T3) were negatively associated with the total femur BMD and the total spine BMD after adjusting for all covariates. Subgroup analyses demonstrated that all groups had a negative association between T4 and BMD, even in patients with osteopenia/osteoporosis. The nonlinear relationship characterized by smooth curve fittings and generalized additive models suggested that an obvious U-shaped, an inverted U -shaped, and an L - shaped curve was exhibited between thyroid hormone and BMD in the different subgroups. In addition, normal high-level T4 was associated with an increased prevalence of previous fractures than normal low-level T4.

**Conclusions:**

In this sample of euthyroid adults, T4 exhibits a negative correlation with BMD, regardless of age and gender, in subjects with either normal or lowered BMD. Moreover, high-normal FT4 was associated with an increased prevalence of previous fractures. TSH was not associated with variations of BMD and the fracture risk.

## 1 Introduction

As the number of osteoporosis individuals increases worldwide, the rate of osteoporotic fracture is rising, resulting in high healthcare costs and tremendous pressure on public health systems (1). The commonly used parameter for bone health evaluation is bone mineral density (BMD). Osteoporosis is accompanied by a decrease in BMD (2, 3). Therefore, it is essential to find out the risk factors for decreased BMD for osteoporosis care and control (4).

The thyroid function, including thyroid -stimulating hormone (TSH), free thyroxine (FT4), total thyroxine (T4), free triiodothyronine (FT3), and total triiodothyronine (T3), has an important impact on skeletal development and bone metabolism. Thyroid dysfunction has detrimental effects on bone structures (5). It is well known that overt hyperthyroidism has a detrimental effect on bone mass and fragility fractures due to a high bone turnover as documented by a shortened bone remodeling cycle, together with an increase in biochemical markers of bone resorption and bone formation (6). In a meta-analysis with 70,298 participants, the patients with subclinical hyperthyroidism were associated with an increased risk of up to 36% of fractures compared to normal people (7). It has been reported that TSH is the negative regulator of bone remodeling by activating the TSH receptors on osteoclast and osteoblast precursors (8, 9). However, due to the different designs and characteristics of the studies, there is a controversy as to the association between TSH and BMD in the euthyroid population (10–13). Acar et al. (14) observed a positive correlation between TSH and BMD among postmenopausal women. However, Zantut-Wittmann et al. (15) found that the thyroid hormone profile was not associated with variations in BMD among euthyroid healthy women who were either normal weight or overweight.

To investigate the influence of thyroid function on BMD among euthyroid adults, we included and analyzed data from the 2007 –2010 cycle of the National Health and Nutrition Examination Survey (NHANES) database to investigate correlates of the thyroid hormone profile (TSH, FT4, T4, FT3, and T3) and BMD in euthyroid adults and determine which of them is more appropriate in studying bone health.

## 2 Materials and methods

### 2.1 Study population

**Figure 1:**
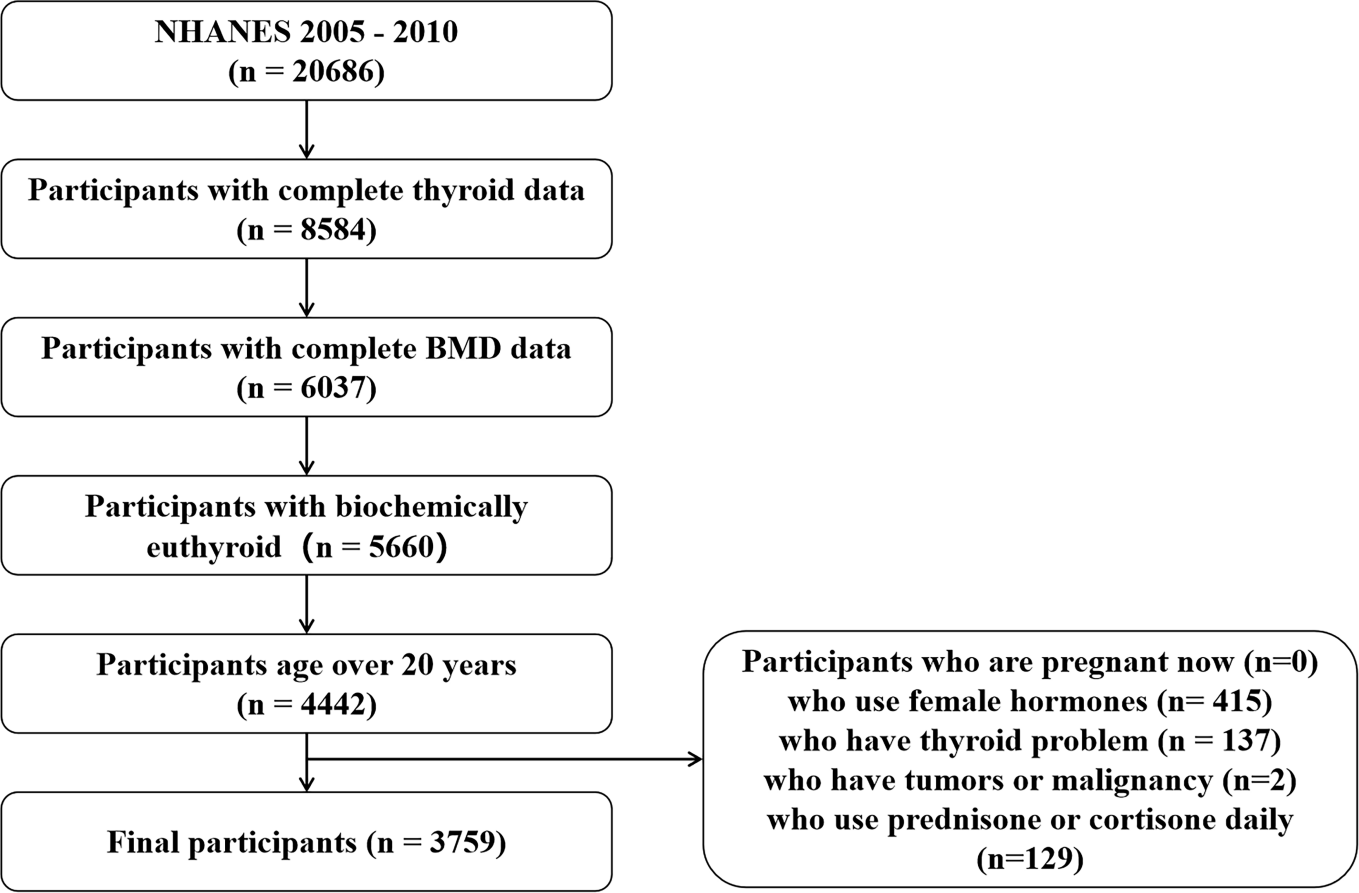
Flow chart of participant selection. NHANES, National Health and Nutrition Examination Survey; BMD, bone mineral density.

The NHANES is a national survey designed to capture nationally representative statistics of US residents and is updated annually. Briefly, a series of sampled household interviews and standardized physical examinations in designated mobile examination centers were arranged across the country. The present study’s data were from two circles of NHANES, from 2007 to 2010. There were 20,686 individuals with laboratory data of the thyroid profile. We defined that the normal ranges for TSH and FT4 were 0.34 –5.60 mIU/L and 0.6 –1.6 ng/dl, respectively (16). There were 4,442 euthyroid adults aged over 20 years with complete data on TSH, FT4, T4, FT3, T3, total femur BMD, and total spine BMD. We excluded all subjects who use female hormones (n = 415), who have thyroid problems (n = 137), who have tumors or malignancy (n = 2), and who use prednisone or cortisone daily (n = 129). Finally, the study population was limited to 3,759 (**Figure 1**). Detailed information on the ethics application and written informed consent is provided on the NHANES website.

### 2.2 Thyroid stimulating hormone (TSH), free thyroxine (FT4), total thyroxine (T4), free triiodothyronine (FT3), and total triiodothyronine (T3)

TSH was quantified with the Access HYPERsensitive human TSH assay, a third-generation two-site immunoenzymatic (“sandwich”) assay. FT4 was quantified with the Access Free T4 assay, a two-step enzyme immunoassay. A competitive binding immunoenzymatic assay was used for T3, FT3, and T4.

### 2.3 Dual X-ray absorptiometry BMD

The BMD was evaluated using the dual-energy X-ray absorptiometry technique [QDR 4500A fan-beam densitometers (Hologic Inc.)] and software version Apex 3.2. The left hip was routinely scanned unless the participant self-reported a fractured left hip, a left hip replacement, or a pin in the left hip to report the total femur BMD. The lumbar spine (L1–L4) was scanned to measure the total spine BMD.

### 2.4 Osteopenia/osteoporosis and fracture history

According to previous studies (17, 18), in women, osteopenia was defined as the total femur BMD value between 0.64 and 0.82 g/cm^2^, and osteoporosis was described as the total femur BMD value < 0.64 g/cm^2^. In men, osteopenia was defined as the total femur BMD value between 0.68 and 0.90 g/cm^2^, and osteoporosis was described as the total femur BMD value < 0.68 g/cm^2^. To determine the self-reported occurrence of osteoporotic fractures, participants were asked, “Has a doctor ever told you that you had a broken or fractured hip/wrist/spine?” and “Has a doctor ever told you that you had broken or fractured any other bone after 20 years of age?”; “yes” answers were recorded as positive.

### 2.5 Other covariates

The following covariates were included in the study: demographic data (gender, age, education, race, and ratio of family income to poverty), hypertension (ever been told you have high blood pressure), diabetes (fasting blood glucose level ≥ 126 mg/dl, a glycated hemoglobin concentration ≥ 6.5%, with prior diagnosis or medical treatment), body mass index (BMI), smoking status, alcohol intake, serum creatine, serum triglyceride, serum total calcium, alkaline phosphatase (ALP), and serum aspartate transaminase (AST). The detailed acquisition process and measuring method of each variable are available at www.cdc.gov/nchs/nhanes.

### 2.6 Statistical analysis

All continuous variables were described as mean (± standard deviation) and categorical variables as percentages (frequency). We checked up dependent variables and residuals with normal tests and homogeneity tests for variance. We analyzed the linear relationship between the thyroid hormone profile (TSH, FT4, T4, FT3, and T3) and BMD of the total femur and spine using weighted multiple regression and their 95% confidence intervals (CIs) among all patients and the patients with osteopenia and osteoporosis. We constructed two distinct models using weighted univariate and multivariate linear regression models, including the unadjusted model (no covariate was adjusted) and the adjusted model (adjusted for gender, age, education, race, ratio of family income to poverty, hypertension, diabetes, BMI, smoking status, alcohol intake, serum creatine, serum triglyceride, serum total calcium, ALP, and AST). Moreover, the nonlinear relationships between the thyroid hormone profile and BMD of the total femur and spine were described by smooth curve fittings and generalized additive models. Subgroup analysis was performed to evaluate relationships in diverse populations by stratifying gender and age (age < 50 years or age ≥ 50 years). We used two-piecewise linear regression models to calculate the inflection point. P values < 0.05 (two-sided) were considered statistically significant. Moreover, logistic regression models were used to determine the association of TSH and T4 (TSH and T4 were divided into a normal low-level group and a normal high-level group by the medians, respectively) with previous fractures. Modeling was performed with the R software v.4.0.3 (Vienna, Austria: R Foundation for Statistical Computing, 2016) and EmpowerStats (version: 2.0; X&Y Solutions, Inc., Boston, MA, USA; http://www.empowerstats.com).

## 3 Results

### 3.1 Patients and baseline characteristics

Overall, we analyzed 3,759 participants [mean age, 44.5 (± 16.2) years; 2,173 (57.8%) men and 1,586 (42.2%) women] in this study. The main education level was above high school [1,737 (46.2%)]. The majority of individuals were non- Hispanic white (43.8%). The ratio of family income to poverty was 2.5 (± 1.6), and the mean BMI was 27.9 (± 5.5) kg/m^2^. Participants with hypertension, diabetes, smoking, and alcohol drinking accounted for 970 (25.8%), 461 (12.3%), 1,728 (46.0%), and 892 (23.7%), respectively. Moreover, the mean of the serum creatine, serum triglyceride, serum total calcium, ALP, and AST was 0.9 (± 0.3) mg/dl, 1.6 ( ± 1.5) mg/dl, 9.4 (± 0.4) mg/dl, 68.3 (± 23.7) U/L, and 26.8 (± 23.2) U/L, respectively. The mean of the thyroid hormone profile (TSH, FT4, T4, FT3, and T3) was 1.7 (± 1.0) mIU/L, 0.8 (± 0.1) ng/dl, 7.8 (± 1.5) µg/dl, 3.2 (± 0.6) pg/ml, and 115.2 (± 22.4) ng/dl, respectively. The mean of total femur BMD among all participants was 1.0 (± 0.16), and the mean of total spine BMD was 1.0 (± 0.15). The detailed results are presented in **Table 1**.

**Table 1:** Characteristics of the participants.

Characteristic	No. reported	Means ± SD or proportions
Prevalence in NHANES (2007–2010)	3,759	
Gender	3,759	
Male	2,173	57.8%
Female	1,586	42.2%
Age (years)	3,759	44.5 ± 16.2
Education level	3,757	
Under high school	1,132	30.1%
High school or equivalent	888	23.6%
Above high school	1,737	46.2%
Race and ethnicity	3,759	
Mexican American	772	20.5%
Other Hispanic	450	12.0%
Non-Hispanic White	1,646	43.8%
Non-Hispanic Black	690	18.4%
Other Race - Including Multiracial	201	5.3%
Ratio of family income to poverty	3,456	2.5 ± 1.6
Hypertension	3,759	
Yes	970	25.8%
No	2,789	74.2%
Diabetes	3,759	
Yes	461	12.3%
No	3,298	87.7%
BMI	3,749	27.9 ± 5.5
Smoking status	3,759	
Yes	1,728	46.0%
No	2,031	54.0%
Alcohol drinking	3,589	
Yes	892	23.7%
No	2,697	71.7%
Serum creatinine (mg/dl)	3,755	0.9 ± 0.3
Serum triglyceride (mmol/L)	1,839	1.6 ± 1.5
Serum total calcium (mg/dl)	3,755	9.4 ± 0.4
ALP (U/L)	3,755	68.3 ± 23.7
AST (U/L)	3,753	26.8 ± 23.2
TSH (mIU/L)	3,759	1.7 ± 1.0
FT4 (ng/dl)	3,759	0.8 ± 0.1
T4 (µg/dl)	3,759	7.8 ± 1.5
FT3 (pg/ml)	3,759	3.2 ± 0.6
T3 (ng/dl)	3,759	115.2 ± 22.4
Total femur BMD	3,759	1.0 ± 0.16
Total spine BMD	3,759	1.0 ± 0.15

BMI, body mass index; ALP, alkaline phosphatase; AST, aspartate aminotransferase; TSH, thyroid -stimulating hormone; FT4, free thyroxine; T4, total thyroxine; FT3, free triiodothyronine; T3, total triiodothyronine; BMD, bone mineral density.

### 3.2 Associations with the total femur BMD and the thyroid hormone profile

#### 3.2.1 Total analysis

**Figure 2:**
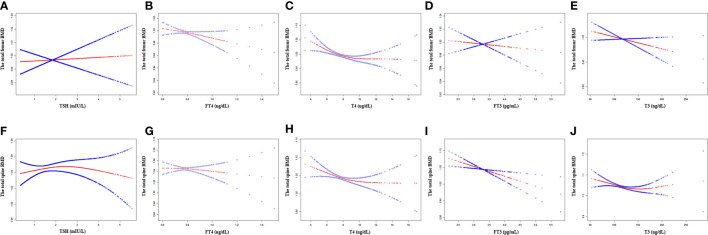
Association between the thyroid hormone profile and BMD. **(A–E)** TSH, FT4, T4, FT3, and T3 with the total femur BMD. **(F–J)** TSH, FT4, T4, FT3, and T3 with the total spine BMD. Solid rad line represents the smooth curve fit between variables. Blue bands represent the 95% confidence interval from the fit. Gender, age, education, race, ratio of family income to poverty, hypertension, diabetes, BMI, smoking status, alcohol intake, serum creatine, serum triglyceride, serum total calcium, ALP, and AST were adjusted.

In the unadjusted model, TSH, FT4, and T4 showed a negative association with the total femur BMD, and FT3 and T3 showed a positive association with the total femur BMD. When adjusting for covariates, FT4, T4, FT3, and T3 still showed a negative association with the total femur BMD. The relationship between TSH and the total femur BMD was not present. Moreover, while smooth curve fittings and generalized additive models were used to characterize the nonlinear relationship, TSH did not show a significant association with the total femur BMD. A negative association was still presented between the other four hormones (FT4, T4, FT3, and T3) and the total femur BMD. The detailed results were shown in **Table 2** and **Figure 2**.

**Table 2:** Association of the thyroid hormone profile with BMD in NHANES, 2007–2010.

	Total femur BMD		Total spine BMD	
	Estimate (95% CI)	P	Estimate (95% CI)	P
Unadjusted model				
**TSH**	-0.007706 (-0.012898, -0.002514)	**0.004**	-0.005922 (-0.010795, -0.001048)	**0.017**
**FT4**	-0.088366 (-0.130263, -0.046470)	**<0.001**	-0.083834 (-0.123141, -0.044527)	**<0.001**
**T4**	-0.009959 (-0.013271, -0.006648)	**<0.001**	-0.007623 (-0.010734, -0.004511)	**<0.001**
**FT3**	0.029045 (0.020261, 0.037830)	**<0.001**	-0.003511 (-0.011799, 0.004776)	0.406
**T3**	0.000377 (0.000155, 0.000600)	**0.001**	-0.000211 (-0.000420, -0.000002)	**0.048**
Adjusted model^a^				
**TSH**	-0.003419 (-0.007637, 0.000800)	0.112	-0.004757 (-0.009381, -0.000132)	0.044
**FT4**	-0.062168 (-0.095733, -0.028603)	**<0.001**	-0.055876 (-0.092702, -0.019050)	**0.003**
**T4**	-0.005419 (-0.008131, -0.002706)	**<0.001**	-0.005047 (-0.008023, -0.002070)	**0.001**
**FT3**	-0.006230 (-0.013643, 0.001183)	**0. 001**	-0.011834 (-0.019957, -0.003711)	**0.004**
**T3**	-0.000315 (-0.000501, -0.000129)	**0.001**	-0.000361 (-0.000565, -0.000157)	**0.001**

BMD, bone mineral density; TSH, thyroid -stimulating hormone; FT4, free thyroxine; T4, total thyroxine; FT3, free triiodothyronine; T3, total triiodothyronine.

a Adjusted for gender, age, education, race, ratio of family income to poverty, hypertension, diabetes, BMI, smoking status, alcohol intake, serum creatine, serum triglyceride, serum total calcium, ALP, and AST. P values less than 0.05 are shown bold.

#### 3.2.2 Subgroup analysis

In subgroup analyses stratified by gender and age, the results showed that there was no association between TSH and the total femur BMD. T4 displayed a negative association with the total femur BMD among all groups.

**Figure 3:**
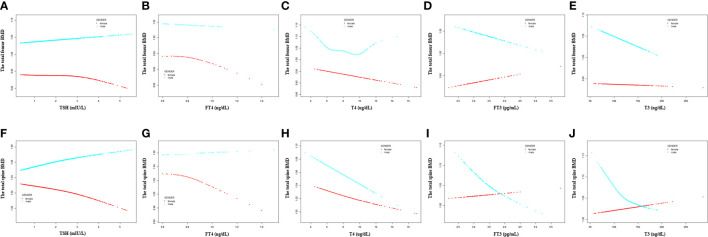
Association between the thyroid hormone profile and BMD stratified by gender. **(A–E)** TSH, FT4, T4, FT3, and T3 with the total femur BMD. **(F–J)** TSH, FT4, T4, FT3, and T3 with the total spine BMD. Solid rad line represents the smooth curve fit between variables. Blue bands represent the 95% confidence interval from the fit. Gender, age, education, race, ratio of family income to poverty, hypertension, diabetes, BMI, smoking status, alcohol intake, serum creatine, serum triglyceride, serum total calcium, ALP, and AST were adjusted.

For men, FT4, T4, FT3, and T3 showed a negative association with the total femur BMD in the adjusted model (**Table 3**). As for women, only FT4 and T4 displayed a negative association with the total femur BMD after adjusting for covariates (**Table 4**). In addition, when the nonlinear relationship was characterized by smooth curve fittings and generalized additive models (**Figure 3**), those negative correlations still survived in most groups. The association between T4 and the total femur BMD was an obvious U-shaped curve among men. The point of inflection identified using a two-piecewise linear regression model was 9.6 µg/dl. A significant negative relationship existed between T4 and the total femur BMD when T4 < 9.6 µg/dl. While the correlation was not significant when T4 > 9.6 µg/dl (**Supplementary Table S1**).

**Table 3:** Association of the thyroid hormone profile with BMD in men.

	Total femur BMD		Total spine BMD	
	Estimate (95% CI)	P	Estimate (95% CI)	P
Unadjusted model				
**TSH**	-0.004852 (-0.011272, 0.001568)	0.138	-0.001747 (-0.008040, 0.004545)	**0.586**
**FT4**	-0.087950 (-0.138325, -0.037574)	**0.001**	-0.071857 (-0.121252, -0.022461)	**0.004**
**T4**	-0.008492 (-0.012807, -0.004178)	**<0.001**	-0.008111 (-0.012338, -0.003883)	**<0.001**
**FT3**	0.036890 (0.020464, 0.053316)	**<0.001**	-0.036754 (-0.052845, -0.020663)	**<0.001**
**T3**	0.000075 (-0.000223, 0.000372)	0.622	-0.000694 (-0.000984, -0.000404)	**<0.001**
Adjusted model^a^				
**TSH**	-0.004657 (-0.010453, 0.001138)	0.115	-0.005517 (-0.011690, 0.000655)	0.080
**FT4**	-0.052851 (-0.097737, -0.007965)	**0.021**	-0.047883 (-0.095710, -0.000057)	**0.049**
**T4**	-0.006840 (-0.010717, -0.002963)	**0.001**	-0.007779 (-0.011907, -0.003651)	**<0.001**
**FT3**	-0.017973 (-0.034790, -0.001155)	**0.036**	-0.030971 (-0.048854, -0.013087)	**0.001**
**T3**	-0.000538 (-0.000818, -0.000259)	**<0.001**	-0.000556 (-0.000854, -0.000258)	**<0.001**

BMD, bone mineral density; TSH, thyroid -stimulating hormone; FT4, free thyroxine; T4, total thyroxine; FT3, free triiodothyronine; T3, total triiodothyronine.

a Adjusted for gender, age, education, race, ratio of family income to poverty, hypertension, diabetes, BMI, smoking status, alcohol intake, serum creatine, serum triglyceride, serum total calcium, ALP, and AST.P values less than 0.05 are shown in bold.

**Table 4:** Association of the thyroid hormone profile with BMD in women.

	Total femur BMD		Total spine BMD	
	Estimate (95% CI)	P	Estimate (95% CI)	P
Unadjusted model				
**TSH**	-0.008796 (-0.016298, -0.001294)	**0.022**	-0.010669 (-0.018259, -0.003079)	**0.006**
**FT4**	-0.133712 (-0.196510, -0.070915)	**<0.001**	-0.115180 (-0.178855, -0.051505)	**<0.001**
**T4**	-0.001042 (-0.005661, 0.003576)	0.658	-0.004224 (-0.008896, 0.000447)	0.076
**FT3**	0.009456 (-0.000319, 0.019231)	0.058	0.002572 (-0.007335, 0.012480)	0.611
**T3**	0.000521 (0.000226, 0.000815)	**0.001**	0.000207 (-0.000092, 0.000506)	0.175
Adjusted model^a^				
**TSH**	-0.000959 (-0.007047, 0.005128)	0.757	-0.000847 (-0.007483, 0.005788)	0.802
**FT4**	-0.070495 (-0.120566, -0.020423)	**0.006**	-0.050782 (-0.105437, 0.003873)	0.069
**T4**	-0.004830 (-0.008558, -0.001101)	**0.011**	-0.004419 (-0.008486, -0.000352)	**0.033**
**FT3**	-0.002083 (-0.009933, 0.005768)	0.603	-0.003701 (-0.012258, 0.004855)	0.396
**T3**	-0.000124 (-0.000367, 0.000119)	0.318	-0.000210 (-0.000474, 0.000055)	0.120

BMD, bone mineral density; TSH, thyroid -stimulating hormone; FT4, free thyroxine; T4, total thyroxine; FT3, free triiodothyronine; T3, total triiodothyronine.

a Adjusted for gender, age, education, race, ratio of family income to poverty, hypertension, diabetes, BMI, smoking status, alcohol intake, serum creatine, serum triglyceride, serum total calcium, ALP, and AST.P values less than 0.05 are shown in bold.

**Figure 4:**
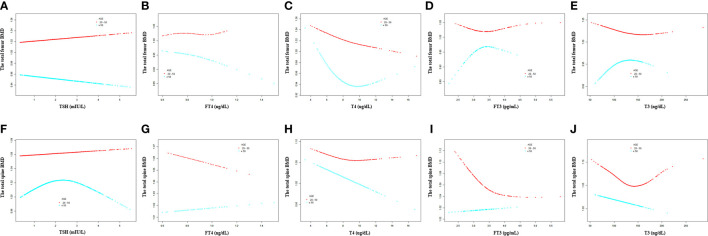
Association between the thyroid hormone profile and BMD stratified by age. **(A–E)** TSH, FT4, T4, FT3, and T3 with the total femur BMD. **(F–J)** TSH, FT4, T4, FT3, and T3 with the total spine BMD. Solid rad line represents the smooth curve fit between variables. Blue bands represent the 95% confidence interval from the fit. Gender, age, education, race, ratio of family income to poverty, hypertension, diabetes, BMI, smoking status, alcohol intake, serum creatine, serum triglyceride, serum total calcium, ALP, and AST were adjusted.

For participants aged 20 –50 years, a negative association was present between T4 and the total femur BMD, but not TSH, FT4, FT3, and T3 (**Table 5**). For participants aged >50 years, FT4 and T4 displayed a negative association with the total femur BMD, and FT3 displayed a positive association with the total femur BMD (**Table 6**). In the smooth curve fittings and generalized additive models (**Figure 4**), FT3 and T3 displayed an inverted U - shaped relationship with the total femur BMD among participants aged from 20 to 50 years with an inflection point of 3 pg/ml and 111 ng/dl, respectively. T4 exhibited an L - shaped relationship with the total femur BMD among participants aged >50 years. In addition, the two-piecewise linear regression models demonstrated that the total femur BMD declined gradually as the T4 level rose (T4 < 6 µg/dl), while no statistical significance was observed when T4 was > 6 µg/dl (**Supplementary Table S2**). FT3 exhibited an inverted U - shaped association with the total femur among participants aged >50 years. The two-piecewise linear regression models demonstrated that the total femur BMD rose gradually with the rising FT3 level (FT3 < 3.37 pg/ml); the total femur BMD declined gradually with the rising FT3 level (FT3 > 3.37 pg/ml).

**Table 5:** Association of the thyroid hormone profile with BMD in participants aged 20 –50 years.

	Total femur BMD		Total spine BMD	
	Estimate (95% CI)	P	Estimate (95% CI)	P
Unadjusted model				
**TSH**	0.002403 (0.003921, 0.008728)	0.456	-0.000692 (-0.006166, 0.004782)	0.804
**FT4**	-0.016875 (-0.066928, 0.033178)	0.509	-0.066439 (-0.109682, -0.023196)	**0.003**
**T4**	-0.006043 (-0.009897, -0.002189)	**0.002**	-0.003547 (-0.006887, -0.000208)	**0.037**
**FT3**	0.010522 (0.001662, 0.019381)	**0.020**	-0.012027 (-0.019688, -0.004365)	**0.002**
**T3**	0.000143 (-0.000109, 0.000395)	0.267	-0.000268 (-0.000486, -0.000050)	**0.016**
Adjusted model^a^				
**TSH**	-0.002366 (-0.007855, 0.003123)	0.398	-0.004514 (-0.009753, 0.000725)	0.091
**FT4**	-0.018814 (-0.062096, 0.024468)	0.394	-0.038450 (-0.079753, 0.002854)	0.0681
**T4**	-0.003792 (-0.007213, -0.000370)	**0.030**	-0.004148 (-0.007414, -0.000883)	**0.013**
**FT3**	-0.002798 (-0.010619, 0.005023)	0.483	-0.006166 (-0.013630, 0.001298)	0.105
**T3**	-0.000122 (-0.000344, 0.000099)	0.279	-0.000167 (-0.000378, 0.000045)	0.123

BMD, bone mineral density; TSH, thyroid -stimulating hormone; FT4, free thyroxine; T4, total thyroxine; FT3, free triiodothyronine; T3, total triiodothyronine.

a Adjusted for gender, age, education, race, ratio of family income to poverty, hypertension, diabetes, BMI, smoking status, alcohol intake, serum creatine, serum triglyceride, serum total calcium, ALP, and AST.P values less than 0.05 are shown in bold.

**Table 6:** Association of the thyroid hormone profile with BMD in participants aged >50 years.

	Total femur BMD		Total spine BMD	
	Estimate (95% CI)	P	Estimate (95% CI)	P
Unadjusted model				
**TSH**	-0.008929 (-0.017619, -0.000238)	**0.044**	-0.005267 (-0.014487, 0.003953)	0.263
**FT4**	-0.170635 (-0.240594, -0.100675)	**<0.001**	-0.093913 (-0.168516, -0.019310)	**0.014**
**T4**	-0.015056 (-0.020832, -0.009280)	**<0.001**	-0.013675 (-0.019812, -0.007537)	**<0.001**
**FT3**	0.075867 (0.048916, 0.102819)	**<0.001**	-0.001746 (-0.030631, 0.027139)	0.906
**T3**	0.000074 (-0.000355, 0.000502)	0.736	-0.000635 (-0.001088, -0.000182)	**0.006**
Adjusted model^a^				
**TSH**	-0.008214 (-0.014933, -0.001494)	0.170	-0.003391 (-0.011461, 0.004678)	0.410
**FT4**	-0.104665 (-0.159139, -0.050191)	**<0.001**	-0.045444 (-0.111038, 0.020151)	0.174
**T4**	-0.006859 (-0.011458, -0.002261)	**0.003**	-0.006729 (-0.012247, -0.001211)	**0.017**
**FT3**	0.031269 (0.009140, 0.053398)	**0.006**	-0.026817 (-0.053378, -0.000256)	**0.048**
**T3**	-0.000089 (-0.000432, 0.000255)	0.612	-0.000516 (-0.000927, -0.000105)	**0.014**

BMD, bone mineral density; TSH, thyroid -stimulating hormone; FT4, free thyroxine; T4, total thyroxine; FT3, free triiodothyronine; T3, total triiodothyronine.

a Adjusted for gender, age, education, race, ratio of family income to poverty, hypertension, diabetes, BMI, smoking status, alcohol intake, serum creatine, serum triglyceride, serum total calcium, ALP, and AST.P values less than 0.05 are shown in bold.

### 3.3 Associations with the total spine BMD and the thyroid hormone profile

#### 3.3.1 Total analysis

As shown in **Table 2**, TSH, FT4, T4, and T3 showed a negative association with the total spine BMD in the unadjusted model; however, the association between TSH and the total spine BMD did not present after adjusting all covariates. FT4, T4, FT3, and T3 displayed a negative association with the total spine BMD in the adjusted model. The fitted curve is demonstrated in **Figure 2**. FT4, T4, FT3, and T3 showed a negative association with the total spine BMD, but not TSH.

#### 3.3.2 Subgroup analysis

In subgroup analyses, the negative association between the thyroid hormone profile and the total spine BMD was mainly present between T4 and the total spine BMD among all groups after adjusting all covariates. There was no association between TSH and the total spine BMD among all groups.

For men, a negative association between FT4, T4, FT3, and T3 with the total spine BMD was displayed after adjusting for all covariates (**Table 3**). As for women, when all covariates were adjusted, only T4 displayed a negative association with the total spine BMD (**Table 4**). When smooth curve fittings and generalized additive models were used to characterize the nonlinear relationship between the thyroid hormone profile and the total spine BMD, those negative associations were still present (**Figure 3**). Moreover, the association between T3 and the total spine BMD was an L - shaped relationship among women. The inflection point was 116 ng/dl. The two-piecewise linear regression models demonstrated that the BMD decreased gradually as the T3 level rose (T3 < 116 ng/dl), while no clear downward trend was observed when T3 > 116 ng/dl (**Supplementary Table S3**).

For participants aged 20 –50 years, a negative association was present between T4 and the total spine BMD, but not TSH, FT4, FT3, and T3 (**Table 5**). For participants aged >50 years, T4, FT3, and T3 displayed a negative association with the total spine BMD (**Table 6**). Moreover, when the nonlinear relationship was characterized by smooth curve fittings and generalized additive models (**Figure 4**), FT3 and T3 displayed an L - shaped relationship with the total spine BMD among participants aged from 20 to 50 years with an inflection point of 3.49 pg/ml and 133 ng/dl, respectively (**Table 4**).

### 3.4 Osteopenia/osteoporosis and fractures

There were 48 participants with osteopenia and 668 participants with osteoporosis. The associations with BMD and the thyroid hormone profile were shown in **Table 7**. T4 displayed a negative association with both the total femur BMD and the spine BMD in the adjusted model. No association between TSH and BMD was observed in both the unadjusted model and the adjusted model.

**Table 7:** Association of the thyroid hormone profile with BMD in participants with osteopenia/osteoporosis.

	Total femur BMD		Total spine BMD	
	Estimate (95% CI)	P	Estimate (95% CI)	P
Unadjusted model				
**TSH**	-0.002765 (-0.008563, 0.003032)	0.349	-0.004385 (-0.013058, 0.004288)	0.321
**FT4**	-0.036604 (-0.081639, 0.008431)	**0.037**	-0.079755 (-0.146999, -0.012511)	**0.020**
**T4**	-0.006219 (-0.010175, -0.002263)	**0.002**	-0.009462 (-0.015379, -0.003545)	0.002
**FT3**	0.048328 (0.032958, 0.063698)	**<0.001**	0.000342 (-0.023260, 0.023944)	0.977
**T3**	0.000349 (0.000082, 0.000617)	0.010	0.000057 (-0.000344, 0.000459)	0.799
Adjusted model ^a^				
**TSH**	0.002144 (-0.002495, 0.006783)	0.365	0.001345 (-0.007422, 0.010111)	0.763
**FT4**	-0.027081 (-0.061874, 0.007713)	0.127	-0.063700 (-0.129350, 0.001950)	0.057
**T4**	-0.003388 (-0.006465, -0.000311)	**0.031**	-0.006647 (-0.012456, -0.000837)	**0.025**
**FT3**	-0.002765 (-0.017002, 0.011473)	0.703	-0.032447 (-0.059230, -0.005664)	**0.018**
**T3**	-0.000052 (-0.000275, 0.000171)	0.646	-0.000026 (-0.000448, 0.000395)	0.903

BMD, bone mineral density; TSH, thyroid -stimulating hormone; FT4, free thyroxine; T4, total thyroxine; FT3, free triiodothyronine; T3, total triiodothyronine.

a Adjusted for gender, age, education, race, ratio of family income to poverty, hypertension, diabetes, BMI, smoking status, alcohol intake, serum creatine, serum triglyceride, serum total calcium, ALP, and AST.P values less than 0.05 are shown in bold.

When logistic regression models were used to determine the association of TSH and T4 with previous fractures, the results showed that TSH was not associated with previous fractures in both the unadjusted analysis [odds ratio (OR) 0.911 (95% CI, 0.788–1.054); P = 0.210] and the multivariate-adjusted analysis [OR 1.097 (95% CI, 0.935–1.287); P = 0.254]. However, in the unadjusted analysis, normal high-level T4 was associated with an increased prevalence of fractures [OR 1.284 (95% CI, 1.109–1.487); P < 0.001]. With the multivariate adjustment, there was still a statistically significant association between normal high-level T4 and previous fracture [OR 1.192 (95% CI, 1.017–1.397); P = 0.031].

## 4 Discussion

In this cross-sectional study, we used the NHANES 2007–2010 database to examine the association between the thyroid hormone profile and BMD (the total femur BMD and the total spine BMD) among euthyroid adults in the US population. We found that there was no obvious association between TSH and BMD. T4 displayed a negative correlation with the total femur BMD and the total spine BMD among all groups. Moreover, a change in the nonlinear relationship between the thyroid hormone profile (FT4, T4, FT3, and T3) and BMD was observed among the different groups.

The human thyroid gland synthesizes, stores, and secretes thyroid hormones, which mainly include 3,5,3′,5′-L-tetraiodothyronine (T4) and a smaller fraction of 3,5,3′-triiodo-l-thyroxine (T3). TSH can bind to its G-protein-coupled receptor in the thyroid stimulation this produces (19). The TSH receptor also expresses in osteoblast (20, 21). T3 and T4 are secreted into the plasma, and most of them are bound to specific proteins. Only a lesser proportion of them exist as the unbound free forms (FT3, FT4) with biological activity and exhibit specific functions at target organs (22). T4 can be converted into T3 with more activity (23). T3 can enter the nucleus and activate either thyroid hormone receptor α or β (TRα, TRβ). TRα is the main receptor expressed in the skeleton (24).

A large number of studies investigating the influence of thyroid disease on the adult skeleton show that thyroid diseases can influence bone health (7, 25, 26). Hyperthyroidism causes high bone turnover with accelerated bone loss and leads to osteoporosis (27). When BMD was evaluated in geriatric patients with toxic nodular goiter, both women and men had a significantly decreased BMD compared to the control (28). Vestergaard and Mosekilde (29) also found that BMD was decreased in untreated patients with hyperthyroidism, with an increased risk of hip fracture, which increases significantly with age. Studies reported that exogenous L-thyroxine, in an inappropriate replacement dose of T4, has a negative effect on bone metabolism (30, 31). Hypothyroidism also can change bone metabolism, resulting in bone mineralization (5). Early histomorphometry analysis demonstrated that hypothyroidism results in low bone turnover with decreased osteoblastic bone formation and reduced osteoclastic bone resorption (24). Some studies suggested that the thyroid status is related to bone health even within the euthyroid reference range, although those results are conflicting (32–35). In the study by Aubert et al. (36), they found that lower TSH and higher FT4 within the reference range were associated with an increased risk of hip fractures. However, van Vliet et al. (37) demonstrated no evidence of a causal effect of circulating TSH on BMD. Van Vliet et al. (37) suggested that in this sample of euthyroid healthy women who were either normal weight or overweight, the thyroid hormone profile was not associated with variations in BMD after a 1-year follow-up. Still, it is a study of a small number of women. In summary, the conclusions remain controversial largely because of heterogeneity, especially with regard to the age and gender of cohorts and differences in study size.

In our study, we used the NHANES database with a representative variety of people and a huge sample size. Considering that previous studies pay more attention to TSH, we analyzed all compositions of thyroid hormones, including FT4, T4, FT3, and T3. After adjusting for variables that might potentially influence BMD, we found that the association between TSH and BMD was not statistically significant, which was consistent with the study of Zantut-Wittmann et al. (15) and Grimnes et al. (33), but in contrast to findings by Lee et al. (38). In the study by Lee et al. (38), they analyzed data from the Korean Urban Rural Elderly (KURE) study and found that the femoral neck and total hip BMD were significantly lower in women with lower normal TSH levels. However, they only focused on older people >65 years old. The differences in ethnicity also could possibly explain part of the discrepancy. The expression of the TSH receptor was discovered in both osteoblasts and osteoclasts, and the receptor activation could regulate cell function (9, 21). It has been reported that subclinical hyperthyroidism (defined as a low TSH level with normal FT4 and FT3 levels) was associated with increased femoral neck bone loss. This may be attributed to the metrological dependence of TSH’s effect on cellular function, which is not evident within the normal range.

Moreover, we found a negative association between thyroid hormones (FT4, T4, FT3, and T3) and BMD. The subgroup analyses, stratified by gender and age, showed that the negative association between T4 and BMD stably existed in different groups. Although the correlation coefficients were small, the inverse association of T4 with BMD was statistically significant in all subgroup analyses and even reached a significance of 0.001 in some analyses. This suggests that there is indeed a weak correlation between T4 and BMD. We think it could be because T4 is the initially synthesized thyroid hormone and the most thyroid hormone in serum, accompanied by an extensive variation range, making it more responsive to the number of thyroid hormones. Thus, T4 could be the most appropriate thyroid function indicator when researching bone and thyroid function among euthyroid adults. We also found consistent results when we investigated patients with osteopenia/osteoporosis. Furthermore, we ran a further exploratory analysis on the associations of TSH and T4 with the history of fractures and found that subjects with normal high-level T4 were more likely to experience fractures. A similar relationship was not found for TSH.

In addition, this study has some limitations. First, this study had a cross-sectional design and only the relationship between thyroid hormone profile and BMD was explored. We could not get a causal inference for the associations between the thyroid hormone profile and bone health or provide any long-term data on the participants. Second, we did not evaluate the direct influence of thyroid hormones on osteoporosis due to the proportion of the osteoporosis participants being too small. Finally, there could be some recall bias in the part of the questionnaires, which might impact the covariates.

## 5 Conclusion

Results of this cross-sectional study demonstrated that in this sample of euthyroid adults, T4 exhibits a negative correlation with BMD, regardless of age and gender, in subjects with either normal or lowered BMD. Moreover, high-normal FT4 was associated with an increased prevalence of previous fractures. TSH was not associated with variations of BMD and the fracture risk. T4 could be a more appropriate indicator than TSH when studying bone and thyroid function. Considering the cross-sectional design of the current study, further prospective and experimental studies are worth conducting to verify our findings and clarify the underlying biological mechanism.

## Data availability statement

The datasets presented in this study can be found in online repositories. The names of the repository/repositories and accession number(s) can be found below: the National Health and Nutrition Examination Survey (NHANES) database.

## Ethics statement

Ethical review and approval was not required for the study on human participants in accordance with the local legislation and institutional requirements. Written informed consent for participation was not required for this study in accordance with the national legislation and the institutional requirements.

## Author contributions

NS and FX contributed to the data collection, analysis, and writing of the manuscript. JW contributed to the analysis and review of the manuscript. ZX contributed to the study design and review of the manuscript. All authors contributed to the development of this manuscript and read and approved the final version.
