# Supplementary material for: T4 rather than TSH correlates with BMD among euthyroid adults
Source: Front Endocrinol (Lausanne). 2023 Jan 9;13:1039079. doi: 10.3389/fendo.2022.1039079 (PMC9868946; doi:10.3389/fendo.2022.1039079)
Supplement: Supplementary file 1 [file DataSheet_1.docx]

**Supplementary material**

Supplementary Table 1. Two-piecewise linear regression models of the thyroid hormone profile on the total femur BMD stratified by gender

| **Gender groups** | | | **Thyroid hormone profile** | | | | |
| --- | --- | --- | --- | --- | --- | --- | --- |
|  | |  | **TSH (mIU/L)** | **FT4 (ng/dL)** | **T4 (ug/dL)** | **FT3 (pg/mL)** | **T3 (ng/dL)** |
| **Male** | |  |  |  |  |  |  |
|  | **Inflection point** | | 3.723 | 1 | 9.6 | 2.9 | 90 |
|  | **< Inflection point** | | 0.000 (-0.011, 0.011) 0.9730 | -0.032 (-0.109, 0.046) 0.4218 | -0.013 (-0.020, -0.006) 0.0006 | 0.106 (-0.066, 0.279) 0.2273 | 0.002 (-0.002, 0.005) 0.3116 |
|  | **> Inflection point** | | 0.034 (-0.009, 0.077) 0.1198 | 0.112 (-0.200, 0.425) 0.4811 | 0.025 (-0.001, 0.051) 0.0646 | -0.019 (-0.047, 0.010) 0.1936 | -0.001 (-0.001, -0.000) 0.0192 |
|  | **Log likelihood ratio** | | 0.166 | 0.404 | **0.011** | 0.17 | 0.185 |
| **Female** | |  |  |  |  |  |  |
|  | **Inflection point** | | 3.914 | 0.079 | 6 | 2.72 | 162 |
|  | **< Inflection point** | | 0.001 (-0.009, 0.011) 0.7702 | 0.079 (-0.070, 0.227) 0.3008 | -0.054 (-0.114, 0.005) 0.0727 | 0.109 (-0.070, 0.288) 0.2331 | -0.000 (-0.001, 0.000) 0.1310 |
|  | **> Inflection point** | | -0.068 (-0.130, -0.006) 0.0309 | -0.146 (-0.266, -0.027) 0.0167 | -0.005 (-0.010, 0.001) 0.0894 | -0.001 (-0.009, 0.007) 0.7976 | 0.001 (-0.001, 0.002) 0.3206 |
|  | **Log likelihood ratio** | | **0.037** | **0.047** | 0.105 | 0.223 | 0.179 |

Abbreviation: TSH, thyroid stimulating hormone; FT4, free thyroxine; T4, total thyroxine; FT3, free triiodothyronine; T3, total triiodothyronine.

Supplementary Table 2. Two-piecewise linear regression models of the thyroid hormone profile on the total femur BMD stratified by age

| Age groups | | | | Thyroid hormone profile | | | | |
| --- | --- | --- | --- | --- | --- | --- | --- | --- |
|  | |  | | TSH (mIU/L) | FT4 (ng/dL) | T4 (ug/dL) | FT3 (pg/mL) | T3 (ng/dL) |
| < 50 years | | |  |  |  |  |  |  |
|  | Inflection point | | | 0.658 | 0.99 | 6 | 3 | 154 |
|  | < Inflection point | | | -0.201 (-0.419, 0.018) 0.0720 | -0.023 (-0.099, 0.052) 0.5445 | -0.039 (-0.082, 0.003) 0.0697 | -0.106 (-0.203, -0.009) 0.0330 | -0.001 (-0.001, -0.000) 0.0090 |
|  | > Inflection point | | | 0.005 (-0.003, 0.013) 0.2427 | 0.171 (-0.189, 0.531) 0.3527 | -0.005 (-0.011, 0.001) 0.0795 | -0.002 (-0.010, 0.007) 0.7195 | 0.001 (-0.000, 0.002) 0.1774 |
|  | Log likelihood ratio | | | 0.065 | 0.326 | 0.125 | **0.036** | **0.043** |
| ≥ 50 years | | |  |  |  |  |  |  |
|  | Inflection point | | | 1.8 | 0.86 | 6 | 3.37 | 93 |
|  | < Inflection point | | | -0.013 (-0.041, 0.016) 0.3752 | 0.032 (-0.097, 0.161) 0.6312 | -0.070 (-0.120, -0.020) 0.0061 | 0.058 (0.010, 0.105) 0.0170 | 0.002 (-0.000, 0.004) 0.1140 |
|  | > Inflection point | | | 0.003 (-0.011, 0.017) 0.6727 | -0.164 (-0.329, 0.002) 0.0530 | -0.001 (-0.008, 0.005) 0.6890 | -0.095 (-0.183, -0.008) 0.0333 | -0.000 (-0.001, 0.000) 0.3928 |
|  | Log likelihood ratio | | | 0.396 | 0.117 | **0.008** | **0.007** | 0.097 |

Abbreviation: TSH, thyroid stimulating hormone; FT4, free thyroxine; T4, total thyroxine; FT3, free triiodothyronine; T3, total triiodothyronine.

Supplementary Table 3. Two-piecewise linear regression models of the thyroid hormone profile on the total spine BMD stratified by gender

| **Gender groups** | | | **Thyroid hormone profile** | | | | |
| --- | --- | --- | --- | --- | --- | --- | --- |
|  | |  | **TSH (mIU/L)** | **FT4 (ng/dL)** | **T4 (ug/dL)** | **FT3 (pg/mL)** | **T3 (ng/dL)** |
| **Male** | |  |  |  |  |  |  |
|  | **Inflection point** | | 1.903 | 0.87 | 8.1 | 3.58 | 116 |
|  | **< Inflection point** | | 0.016 (-0.006, 0.038) 0.1567 | 0.047 (-0.068, 0.161) 0.4227 | -0.017 (-0.028, -0.005) 0.0036 | -0.051 (-0.092, -0.009) 0.0182 | -0.002 (-0.003, -0.001) 0.0005 |
|  | **> Inflection point** | | -0.006 (-0.021, 0.010) 0.4724 | -0.050 (-0.226, 0.126) 0.5767 | -0.003 (-0.016, 0.010) 0.6834 | -0.009 (-0.064, 0.046) 0.7516 | -0.000 (-0.001, 0.001) 0.6550 |
|  | **Log likelihood ratio** | | 0.183 | 0.44 | 0.18 | 0.296 | **0.031** |
| **Female** | |  |  |  |  |  |  |
|  | **Inflection point** | | 3.162 | 0.77 | 8.3 | 2.98 | 161 |
|  | **< Inflection point** | | 0.007 (-0.007, 0.022) 0.3034 | 0.117 (-0.089, 0.323) 0.2651 | -0.009 (-0.022, 0.004) 0.1805 | -0.085 (-0.176, 0.006) 0.0675 | -0.000 (-0.001, 0.000) 0.1040 |
|  | **> Inflection point** | | -0.032 (-0.068, 0.005) 0.0878 | -0.079 (-0.214, 0.055) 0.2486 | -0.000 (-0.010, 0.009) 0.9662 | -0.002 (-0.011, 0.008) 0.7251 | 0.001 (-0.001, 0.002) 0.2229 |
|  | **Log likelihood ratio** | | 0.083 | 0.181 | 0.356 | 0.072 | 0.114 |

Abbreviation: TSH, thyroid stimulating hormone; FT4, free thyroxine; T4, total thyroxine; FT3, free triiodothyronine; T3, total triiodothyronine.

Supplementary Table 4. Two-piecewise linear regression models of the thyroid hormone profile on the total spine BMD stratified by age

| Age groups | | | | Thyroid hormone profile | | | | |
| --- | --- | --- | --- | --- | --- | --- | --- | --- |
|  | |  | | TSH (mIU/L) | FT4 (ng/dL) | T4 (ug/dL) | FT3 (pg/mL) | T3 (ng/dL) |
| < 50 years | | |  |  |  |  |  |  |
|  | Inflection point | | | 2.132 | 0.7 | 8.3 | 3.49 | 133 |
|  | < Inflection point | | | 0.007 (-0.010, 0.023) 0.4302 | 0.116 (-0.153, 0.385) 0.3990 | -0.013 (-0.023, -0.004) 0.0059 | -0.050 (-0.086, -0.014) 0.0065 | -0.001 (-0.001, -0.000) 0.0035 |
|  | > Inflection point | | | -0.004 (-0.019, 0.012) 0.6427 | -0.036 (-0.113, 0.042) 0.3664 | 0.001 (-0.008, 0.011) 0.7836 | -0.002 (-0.011, 0.007) 0.6504 | 0.000 (-0.000, 0.001) 0.2993 |
|  | Log likelihood ratio | | | 0.458 | 0.319 | 0.062 | **0.013** | **0.022** |
| ≥ 50 years | | |  |  |  |  |  |  |
|  | Inflection point | | | 1.758 | 0.79 | 10.6 | 2.71 | 106 |
|  | < Inflection point | | | 0.035 (-0.002, 0.072) 0.0639 | 0.128 (-0.086, 0.341) 0.2423 | -0.008 (-0.018, 0.001) 0.0915 | -0.143 (-0.418, 0.133) 0.3109 | -0.001 (-0.003, 0.001) 0.2032 |
|  | > Inflection point | | | -0.011 (-0.028, 0.007) 0.2283 | -0.029 (-0.184, 0.126) 0.7133 | 0.018 (-0.016, 0.052) 0.2968 | -0.021 (-0.068, 0.025) 0.3718 | 0.000 (-0.001, 0.001) 0.9422 |
|  | Log likelihood ratio | | | 0.057 | 0.312 | 0.165 | 0.402 | 0.308 |

Abbreviation: TSH, thyroid stimulating hormone; FT4, free thyroxine; T4, total thyroxine; FT3, free triiodothyronine; T3, total triiodothyronine.
